# Collagen IV of basement membranes: II. Emergence of collagen IV^α345^ enabled the assembly of a compact GBM as an ultrafilter in mammalian kidneys

**DOI:** 10.1016/j.jbc.2023.105459

**Published:** 2023-11-15

**Authors:** Elena N. Pokidysheva, Neve Redhair, Octavia Ailsworth, Patrick Page-McCaw, Louise Rollins-Smith, Vijayishwer Singh Jamwal, Yuko Ohta, Hans Peter Bächinger, Prayag Murawala, Martin Flajnik, Agnes B. Fogo, Dale Abrahamson, Julie K. Hudson, Sergei P. Boudko, Billy G. Hudson

**Affiliations:** 1 Division of Nephrology and Hypertension, Department of Medicine, Vanderbilt University Medical Center, Nashville, Tennessee, USA; 2 Aspirnaut, Vanderbilt University Medical Center, Nashville, Tennessee, USA; 3 Center for Matrix Biology, Vanderbilt University Medical Center, Nashville, Tennessee, USA; 4 Department of Pathology Microbiology and Immunology, Vanderbilt University School of Medicine, Nashville, Tennessee, USA; 5 Mount Desert Island Biological Laboratory, Bar Harbor, Maine, USA; 6 Department of Microbiology and Immunology, School of Medicine, University of Maryland, Baltimore, Maryland, USA; 7 Research Department, Shriners Hospital for Children, Portland, Oregon, USA; 8 Clinic for Kidney and Hypertension Diseases, Hannover Medical School, Hannover, Germany; 9 Department of Pathology, Microbiology and Immunology, Vanderbilt University Medical Center, Nashville, Tennessee, USA; 10 Department of Cell Biology and Physiology, The Jared Grantham Kidney Institute, University of Kansas Medical Center, Kansas City, Kansas, USA; 11 Department of Biochemistry, Vanderbilt University, Nashville, Tennessee, USA; 12 Vanderbilt-Ingram Cancer Center, Vanderbilt University, Nashville, Tennessee, USA; 13 Vanderbilt Institute of Chemical Biology, Vanderbilt University, Nashville, Tennessee, USA; 14 Department of Biological Sciences, Vanderbilt University, Nashville, Tennessee, USA; 15 Department of Cell and Developmental Biology, Vanderbilt University, Nashville, Tennessee, USA

**Keywords:** collagen IV, glomerular basement membrane, NC1 domain, ultrafiltration, protein evolution, extracellular matrix, glomerular filtration barrier, Alport syndrome, Goodpasture disease, chronic kidney disease

## Abstract

The collagen IV^α345^ (Col-IV^α345^) scaffold, the major constituent of the glomerular basement membrane (GBM), is a critical component of the kidney glomerular filtration barrier. In Alport syndrome, affecting millions of people worldwide, over two thousand genetic variants occur in the *COL4A3*, *COL4A4*, and *COL4A5* genes that encode the Col-IV^α345^ scaffold. Variants cause loss of scaffold, a suprastructure that tethers macromolecules, from the GBM or assembly of a defective scaffold, causing hematuria in nearly all cases, proteinuria, and often progressive kidney failure. How these variants cause proteinuria remains an enigma. In a companion paper, we found that the evolutionary emergence of the *COL4A3*, *COL4A4*, *COL4A5,* and *COL4A6* genes coincided with kidney emergence in hagfish and shark and that the *COL4A3* and *COL4A4* were lost in amphibians. These findings opened an experimental window to gain insights into functionality of the Col-IV^α345^ scaffold. Here, using tissue staining, biochemical analysis and TEM, we characterized the scaffold chain arrangements and the morphology of the GBM of hagfish, shark, frog, and salamander. We found that α4 and α5 chains in shark GBM and α1 and α5 chains in amphibian GBM are spatially separated. Scaffolds are distinct from one another and from the mammalian Col-IV^α345^ scaffold, and the GBM morphologies are distinct. Our findings revealed that the evolutionary emergence of the Col-IV^α345^ scaffold enabled the genesis of a compact GBM that functions as an ultrafilter. Findings shed light on the conundrum, defined decades ago, whether the GBM or slit diaphragm is the primary filter.

Chronic kidney disease (CKD), including acquired and genetic forms, affects more than 10% of the world’s population (2, 3, 4, 5, 6). The diagnosis is made based on one of the criteria: low glomerular filtration rate and persistent proteinuria (7). In many cases, the glomerular filtration barrier becomes dysfunctional, which leads to leakage of protein across the barrier into the filtrate. A suggested mechanism is that excessive leaked protein is then reabsorbed by the proximal tubules causing tubulointerstitial inflammation and scarring, resulting in progressive kidney failure (8, 9). Proteinuria is the hallmark feature of CKD (10, 11, 12). The pathologies include diabetic nephropathy (13, 14), Alport syndrome (5, 15), Pierson syndrome (16, 17, 18), minimal change disease (19), membranous nephropathy (20), lupus nephritis (21, 22), focal segmental glomerulosclerosis (23), and congenital nephrotic syndrome of Finnish type (24, 25).

Over several decades, numerous searches of pathogenic mechanisms in a few diseases have identified the glomerular basement membrane (GBM), podocytes, and slit diaphragm as critical components of the filtration barrier (6, 12, 26). The pivotal advances were the discovery of proteins that are linked to filtration dysfunction. These are Col-IV^α345^ scaffold in Goodpasture disease (27, 28, 29, 30, 31) and Alport syndrome (31, 32, 33, 34), laminin-521 in Pierson syndrome (17, 35, 36), nephrin in congenital nephropathy (24, 37), and PLA2R in membranous nephropathy (38). Yet, the molecular mechanisms of how these proteins enable ultrafiltration and how genetic variants cause dysfunction remain largely unknown.

The critical function of the GBM in ultrafiltration was illuminated by discoveries of proteins and defects in their structures. The Col-IV^α345^ scaffold is the major constituent of GBM accounting for greater than 70% of its mass† (31, 39, 40, 41, 42, 43, 44). According to the LOVD database, in Alport syndrome, affecting millions of people worldwide, over two thousand genetic variants are known in the *COL4A3*, *COL4A4*, and *COL4A5* genes that encode the α3, α4 and α5 chains of the Col-IV^α345^ scaffold, and this number is growing (4, 5, 45, 46, 47) (Fig. 1). Pathogenic variants cause either loss of Col-IV^α345^ scaffold from the GBM or assembly of a defective scaffold, causing hematuria and often progressive proteinuria and kidney failure (45). Proteinuria is more common in the X-linked and autosomal recessive forms of Alport syndrome which constitute the majority of the diagnosed cases, while clinical presentations of patients with autosomal dominant inheritance varies widely (48). In Pierson syndrome, genetic variants of laminin-521 network of GBM cause acute proteinuria and kidney failure (35, 49). How these variants cause proteinuria posits a fundamental unanswered question: **“How do Col-IV**^**α345**^ **and laminin-521 enable GBM to function as an ultrafilter of proteins?”**

**Figure 1:**
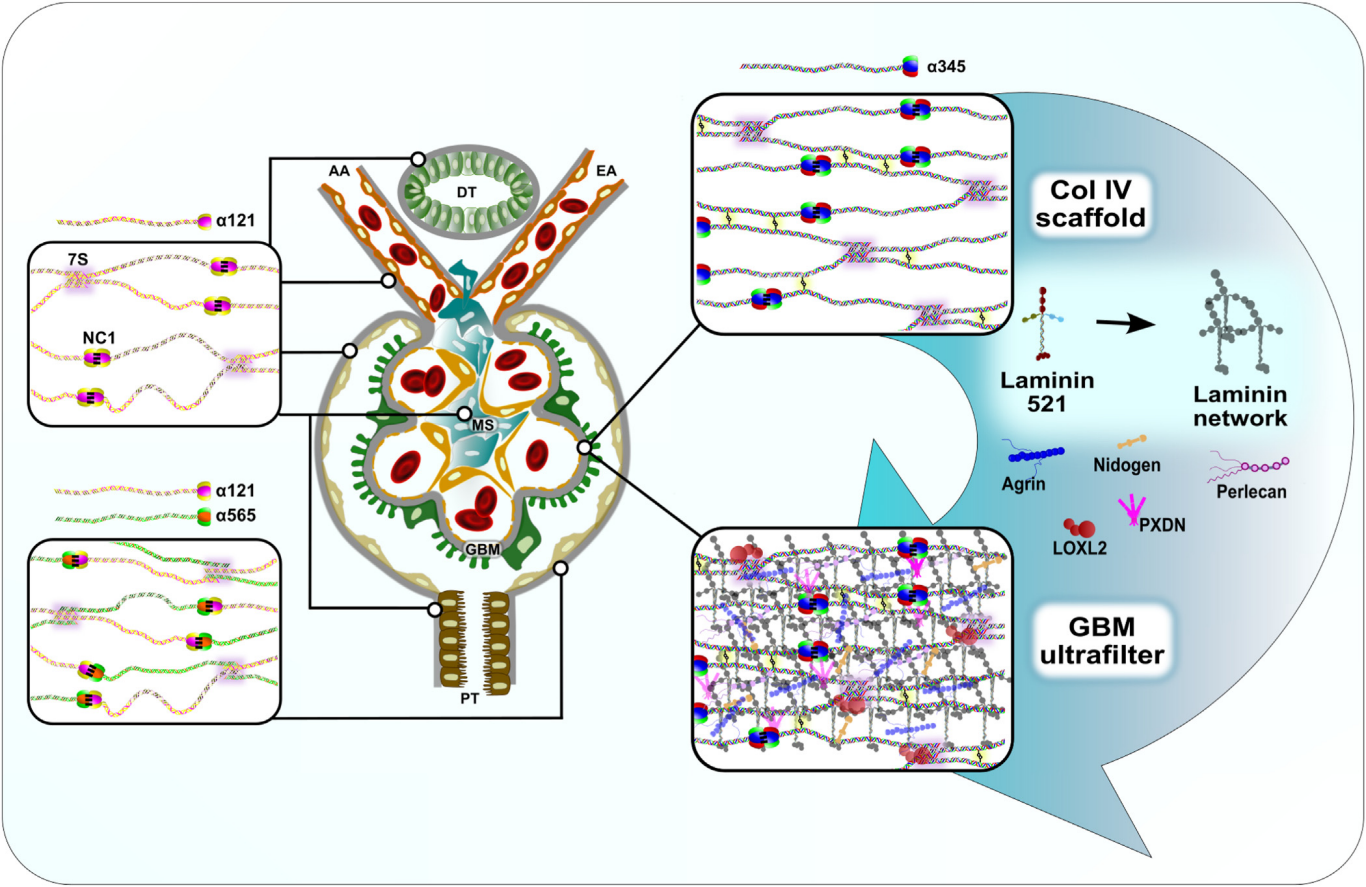
**Col-IV scaffold compositions and localizations in a portion of a mammalian nephron.** The Col-IV^α345^ scaffold encodes information for the tethering of GBM macromolecules. A cross-section of a portion of a nephron is schematically represented with the following designations: **DT** – distal tubule; **PT** – proximal tubule; **MS** – mesangial space; **AA** – afferent arteriole; **EA** – efferent arteriole; **GBM** – glomerular basement membrane. The **NC1**- and **7S**-domains (indicated for Col-IV ^α121^ scaffold) direct the assembly of protomers into network structures of higher order stabilized by sulfilimine bonds on one side (**NC1**, *black stitches*) and lysyl crosslinks on the other (**7S**, highlighted). There are also lateral interactions of collagen triple helices which are not represented here (80). The resulting meshwork sizes of the scaffolds are smaller than shown. The Col-IV^α121^ scaffold is the key building block of the basement membranes surrounding tubules, arterioles and Bowman’s capsule. It is also found in the mesangial area. Bowman’s capsule is a unique structure containing heteroscaffold of Col-IV^α121-α565^. The Col-IV^α345^ scaffold (on the *right*)—a basis of GBM suprastructure—is also reinforced by disulfide bonds (highlighted with *yellow*). Once assembled, Col-IV networks function as smart scaffolds. Vast amounts of structural information are encoded in motifs located at specific sites along the triple helix and NC1 domains to tether other GBM components such as **laminin**-**521**, **nidogen**, **perlecan**, and **agrin**. Laminin also forms network that becomes closely associated with Col-IV^α345^ scaffold. Two important enzymes, **PXDN** and **LOXL2**, crosslink the Col-IV^α345^ scaffold. The tethering at specific sites spatially organizes molecules along the triple helix, resulting in a populated scaffold within the GBM that provides tensile strength and allows for GBM to filter proteins.

Our findings on the evolutionary origin and diversification of collagen IV genes described in details in a companion paper by McCaw *et al.* (50) opened an experimental window to gain molecular insights into the functionality of the Col-IV^α345^ scaffold. In summary, we found that the *COL4A3* and *COL4A4* gene pair appeared in cyclostomes (hagfish and lampreys), while the *COL4A5* and *COL4A6* gene pair emerged in gnathostomes, jawed vertebrates (sharks). We also demonstrated in this study that all six chains of collagen IV were present in the shark kidney. The more basal chordate species, lancelets and tunicates, do not have discrete kidneys and have a single *COL4A* gene pair. While the six *COL4* genes are conserved in vertebrates, amphibians have lost the *COL4A3* and *COL4A4* genes. Here, we characterized the scaffold arrangement of Col-IV chains and the GBM morphology of hagfish, shark, frog, and salamander. We describe the functionality of Col-IV^α345^ in GBM assembly and ultrafiltration and shed light on the conundrum, defined 4 decades ago, as to the nature of the primary protein filter, the GBM or the slit diaphragm (51, 52, 53).

## Results

### Col-IV scaffold organization of α-chains in shark kidney is distinct from mammals

**Figure 2:**
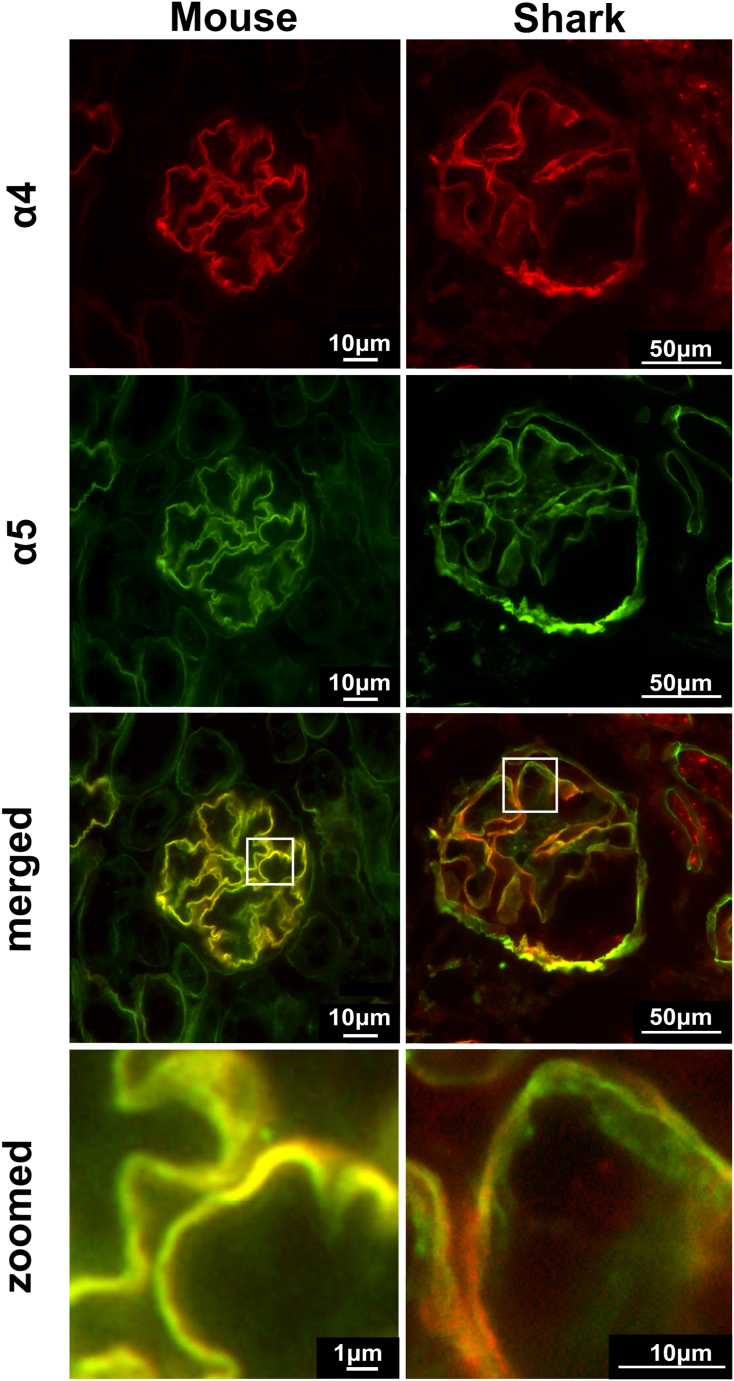
**Col IV α4 and α5 chains have distinct distributions in shark GBM.** Double immunofluorescent staining for α4 (*red*) and α5 (*green*) chains in mouse and shark kidney sections. Glomeruli are represented. Both Col-IV chains localizing to the GBM in mouse and shark. Two *bottom panels* show merged images and zoomed regions indicated by *squares*. *Yellow color* in the merged and zoomed images indicates colocalization of two chains. No *red color* is observed in the merged image of mouse GBM. Two separate layers of green and red staining can be distinguished in the merged image of shark GBM (*green* and *red colors* in zoomed image). GBM, glomerular basement membrane.

In McCaw *et al.* (50), we demonstrated that all six Col-IV chains (α1 to α6) emerged in the *jawed vertebrates (gnathostomes)* and are expressed in shark kidneys. These findings raised the question of whether the α3, α4, and α5 chains of shark kidney occur in a Col-IV^α^^345^ scaffold, analogous to that of mammals. To this end, we performed a double immunofluorescence analysis of shark kidney sections in comparison with control mouse kidney sections. We observed that α4 and α5 collagen IV chains are present in the glomeruli of both mouse and shark. In mouse, these chains colocalize completely in the glomerular basement membrane. Yellow color throughout the GBM in the merged and zoomed images of the mouse glomerulus indicates that both chains are part of the α345 scaffold (Fig. 2). The α4 chain is only known to exist as part of the α345 protomer (54); therefore, no red staining is seen in the merged image for mouse. In contrast, α5 chain is a component of two protomers: α345 and α556 with different tissue specificity as previously reported (55); this distribution is evident from the green staining of tubules and Bowman’s capsule for α5 and where α4 is not present (Fig. 2, green staining in merged image for mouse). These findings demonstrate the presence of the mammalian Col-IV^α^^345^ scaffold and serve as a control for the analysis of shark kidney.

**Figure 3:**
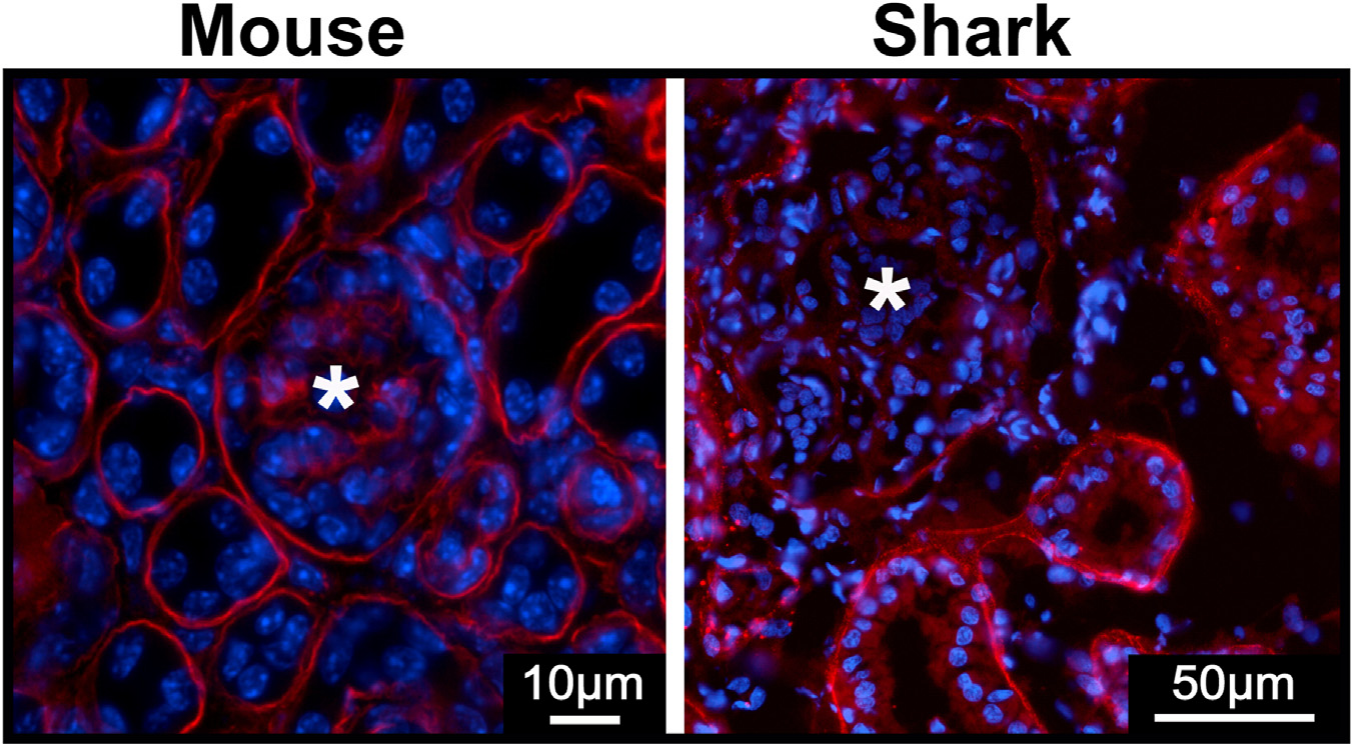
**Col-IV α1 chain is found in tubular basement membrane and Bowman’s capsule but not in glomerulus of the shark.** Immunofluorescent staining for α1 chain of collagen IV in mouse and shark kidney sections. “**∗**” indicates glomerulus. The α1 is found in the Bowman’s capsule surrounding glomerulus and tubular basement membranes as well as mesangial space of the mouse. No staining is present in the mesangial matrix and GBM of the shark. GBM, glomerular basement membrane.

In striking contrast, the distribution of α4 and α5 chains in the shark is distinctly different from the mouse. Figure 2 shows that both chains are found within the capillary loops of shark glomerulus. However, very little colocalization is seen in the merged image. Zoomed image shows magnified view of the capillary loop. Red and green staining indicate that α4 and α5 collagen chains are spatially separated. These staining patterns suggest that the mammalian Col-IV^α^^345^ scaffold is absent in shark kidney. Interestingly, the Col-IV^α^^121^ scaffold is absent in shark glomerulus but present in Bowman’s capsule and tubules (Fig. 3).

**Figure 4:**
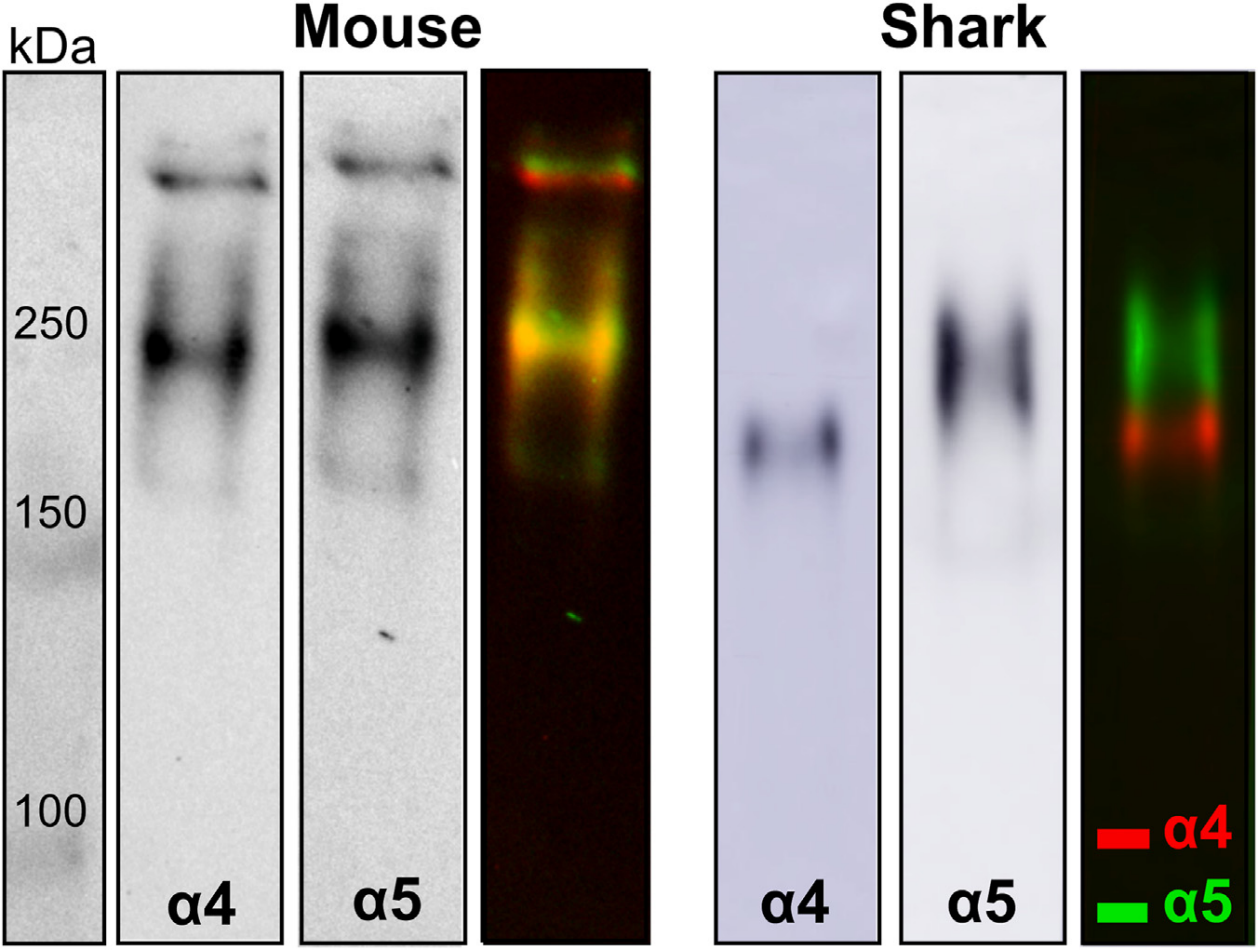
**Col-IV α4 and α5 chains occur in different hexamers in shark kidney.** Mouse and shark kidney samples treated with collagenase to release the NC1 hexamer domains were separated by native PAGE electrophoresis and blotted. Western blots were probed consecutively with α4- and α5-specific antibodies. Third panel for each specie represents artificially colored overlaid image. The positions of α4 and α5 containing NC1 hexamers overlap in the mouse (*yellow color* in the third mouse panel) but separate in the shark (*green and red* in the *right panel* for shark). GBM, glomerular basement membrane.

To further explore the scaffold organization of α4 and α5 chains in shark, we characterized the chain compositions of NC1 hexamers. Hexamers, which reflect the organization of chains, were excised from kidney by collagenase digestion, and their compositions were analyzed by native PAGE. Due to differences in the amino acid sequences/charges hexamers of varying chain composition run differently on the native gel. We performed a comparative native PAGE analysis of hexamers excised from mouse and shark kidneys. Consecutive western blots with α4 and α5 chain-specific antibodies were performed on the same membrane with a stripping step in between. Positions of the hexamer bands positive for α4 and α5 chains were compared by overlaying the western blots (Fig. 4). Western blots for each chain and overlaid, artificially colored image, reveal that α4 and α5 NC1 domains occur in the same hexamer in mouse but in two distinct hexamer populations in shark kidney, Collectively, staining patterns and native PAGE analysis indicate that the Col-IV^α^^345^ scaffold is absent in shark kidney. Thus, other arrangements of the α4 and α5 chains comprise shark GBM.

### Col-IV scaffold organization of α-chains in amphibian kidney is distinct from sharks and mammals

**Figure 5:**
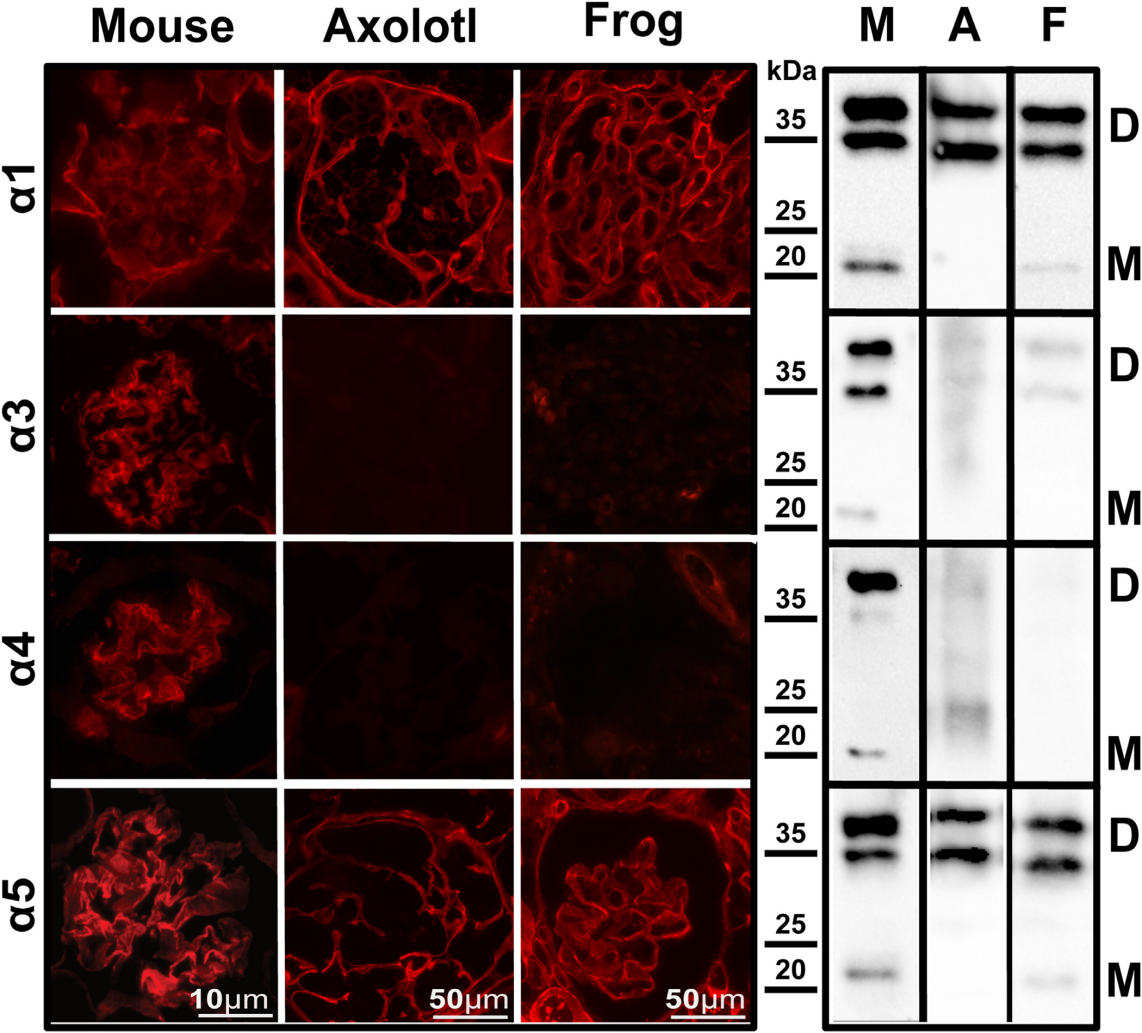
**Col-IV chains distribution in amphibian kidneys.** Immunofluorescence staining of mouse, frog, and axolotl kidney sections (*left*) demonstrates deposition of α1 and α5 chains within Col-IV scaffolds. Glomeruli in each species are represented. No staining is detected for α3 and α4 chains in amphibians. *Right* part of the figure shows western blots of the whole kidney homogenates subjected to the collagenase digest. Mouse (**M**), axolotls (**A**) and frogs (**F**) are shown. On the *right-side* “**M**” indicates monomers and “**D**” – dimers of Col-IV NC1 domains.

As we described in the companion paper by McCaw *et al.* (50), the *COL4A3* and *COL4A4* genes were lost in amphibians. Thus, the Col-IV scaffold of amphibians, which represents naturally occurring double knockouts of α3 and α4 chains, is distinct from the Col-IV^α^^345^ scaffold of mammals. We investigated the tissue distribution of collagen IV chains expressed in the kidneys of axolotl (*Ambystoma mexicanum*) and frog (*Xenopus laevis*). Kidney sections and whole kidney homogenates were analyzed by immunofluorescence and Western blotting with chain-specific antibodies (Fig. 5). The results show the absence of α3 and α4 chains in the glomeruli of both species and the presence of α1 and α5 chains. In contrast to mouse, staining for α1 chain is prominent in the glomerular filtration barrier of both amphibians. These differences between species indicate that the Col-IV scaffold of amphibian glomeruli is distinct from mammals with respect to chain composition.

**Figure 6:**
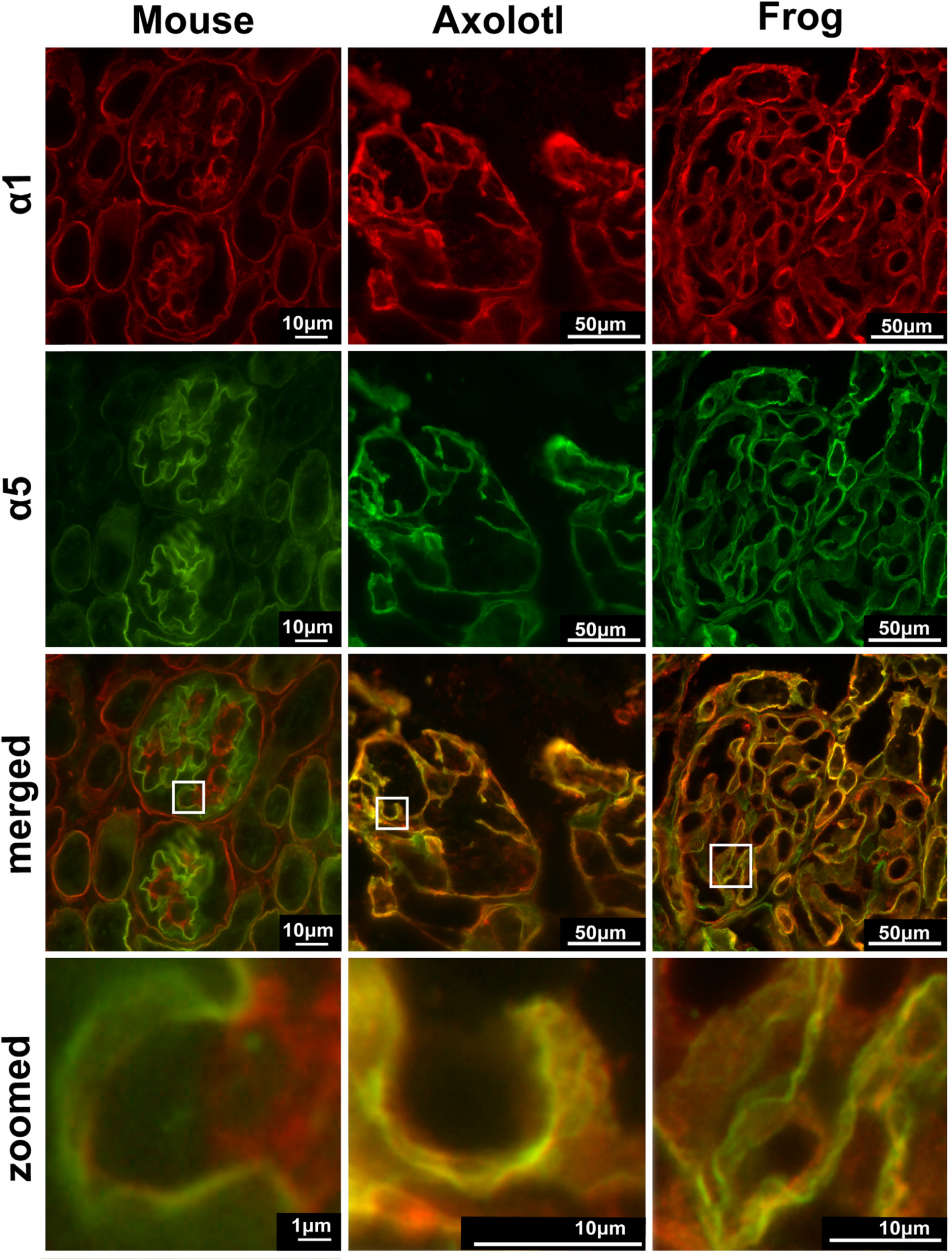
**Amphibian α1 and α5 chains of collagen IV are spatially close but distinct in glomerular capillary loops.** Double immunofluorescent staining for α1 (*red*) and α5 (*green*) chains in mouse; axolotl; and frog kidney sections. The image for α1 staining is reproduced from Fig. 5, Frog, for comparison. *Bottom panels* represent merged and zoomed images of squared regions. *Yellow color* in the merged, and zoomed images indicates colocalization of two chains. While some colocalization is observed in the amphibians, at some places two separate layers of *green* and *red* staining can be distinguished (zoomed images for axolotl and frog).

We explored the molecular distribution of Col-IV α chains by double immunofluorescence staining for α1 and α5 chains in kidneys of axolotl and frog and compared it to mouse kidney. In mouse glomerulus, the α1 chain occurs mostly in the mesangial matrix and the α5 chain in the glomerular capillary loops (Fig. 6). Whereas, in amphibians, the α5 occurs along with α1 in capillary loops; in the merged images, partial colocalization of α1 and α5 chains is evident. However, magnified views show that these chains form two spatially close, but distinct layers (red and green staining in zoomed panel of Fig. 6). These results suggest that the α1 and α5 chains are spatially separated in amphibians and therefore exist in two different Col-IV scaffolds.

**Figure 7:**
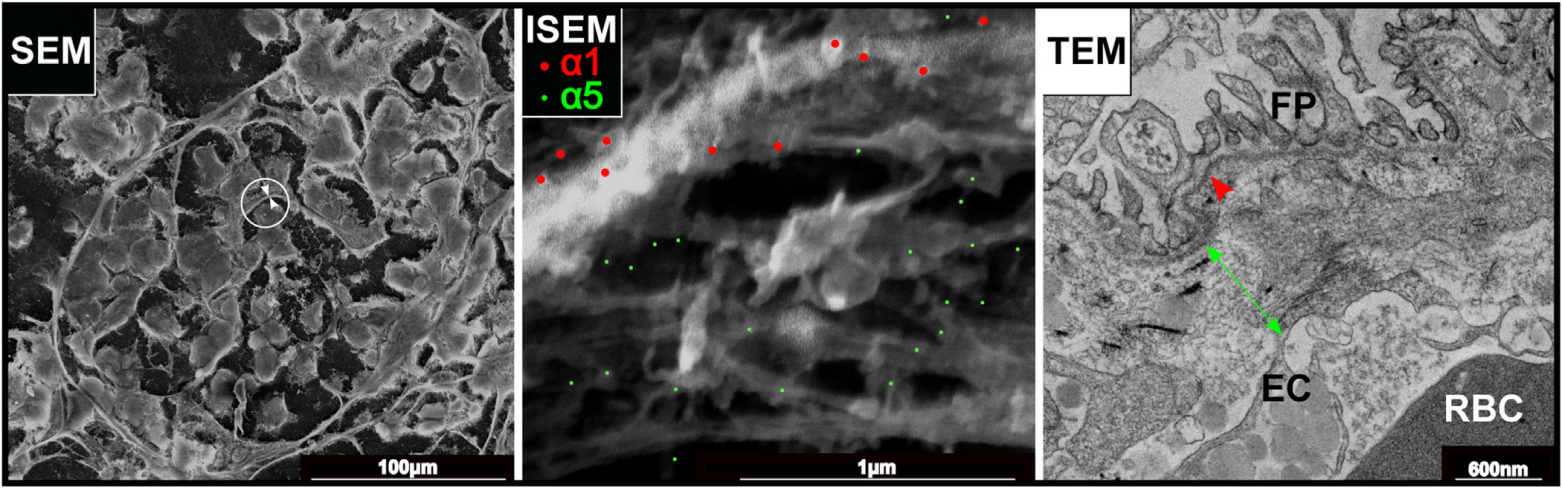
**Col-IV α1 and α5 chains occur in two distinct scaffolds in glomerular filtration barrier of axolotl.** Scanning electron microscopy (SEM) and transmission electron microscopy (TEM) images of the kidney sections from axolotl. The image of the whole glomerulus by SEM is shown on the *left*. Middle image of ISEM shows magnified view of the capillary loop (circled in the SEM image) double immunolabeled with α1 and α5 chain-specific collagen IV antibodies. The ISEM image (*middle*) represents overlay of secondary and backscattered electron images with artificially colored gold particles. Secondary antibodies conjugated with 15 nm (*red*, α1) and 6 nm (*green*, α5) gold particles were used. The TEM image (*right*) demonstrates morphology of the glomerular filtration unit in the axolotl. “**FP**” indicates foot processes of podocytes; “**EC**” designates endothelial cells, and “**RBC**” stands for red blood cell inside the capillary loop. *Double headed green arrow* shows thick, electron lucent portion of GBM which correlates with α5-positive layer in ISEM. *Red arrowhead* points to the thin electron dense layer of GBM underlining podocytes which mainly contains α1 in ISEM. GBM, glomerular basement membrane; ISEM, immunogold labeling and scanning electron microscopy.

For verification of this distribution in the axolotl kidney, we performed double immunogold labeling for analysis at the nanoscale level, using scanning electron microscopy (ISEM). We used 15 nm and 6 nm gold particles to distinguish between α1 and α5 chains, respectively. Figure 7 demonstrates that both types of gold particles are found in the capillary loop matrix with spatially distinct labeling patterns. The α1 labeling (red circles in the middle image in Fig. 7) is restricted to the thin compact layer close to the podocytes. When correlated with the TEM image, an electron dense layer of basement membrane underlying the podocytes (red arrowhead in TEM image) consists predominantly of Col-IV α1 and sparse matrix below that layer is positive for the α5 chain (green double headed arrow). Collectively, the results indicate that α1 and α5 chains comprise two distinct Col-IV scaffolds, spaced between podocytes and endothelial cells, making a “double layered” glomerular basement membrane.

### Amphibian Col-IV α1 and α5 chains comprise two different NC1 hexamers

**Figure 8:**
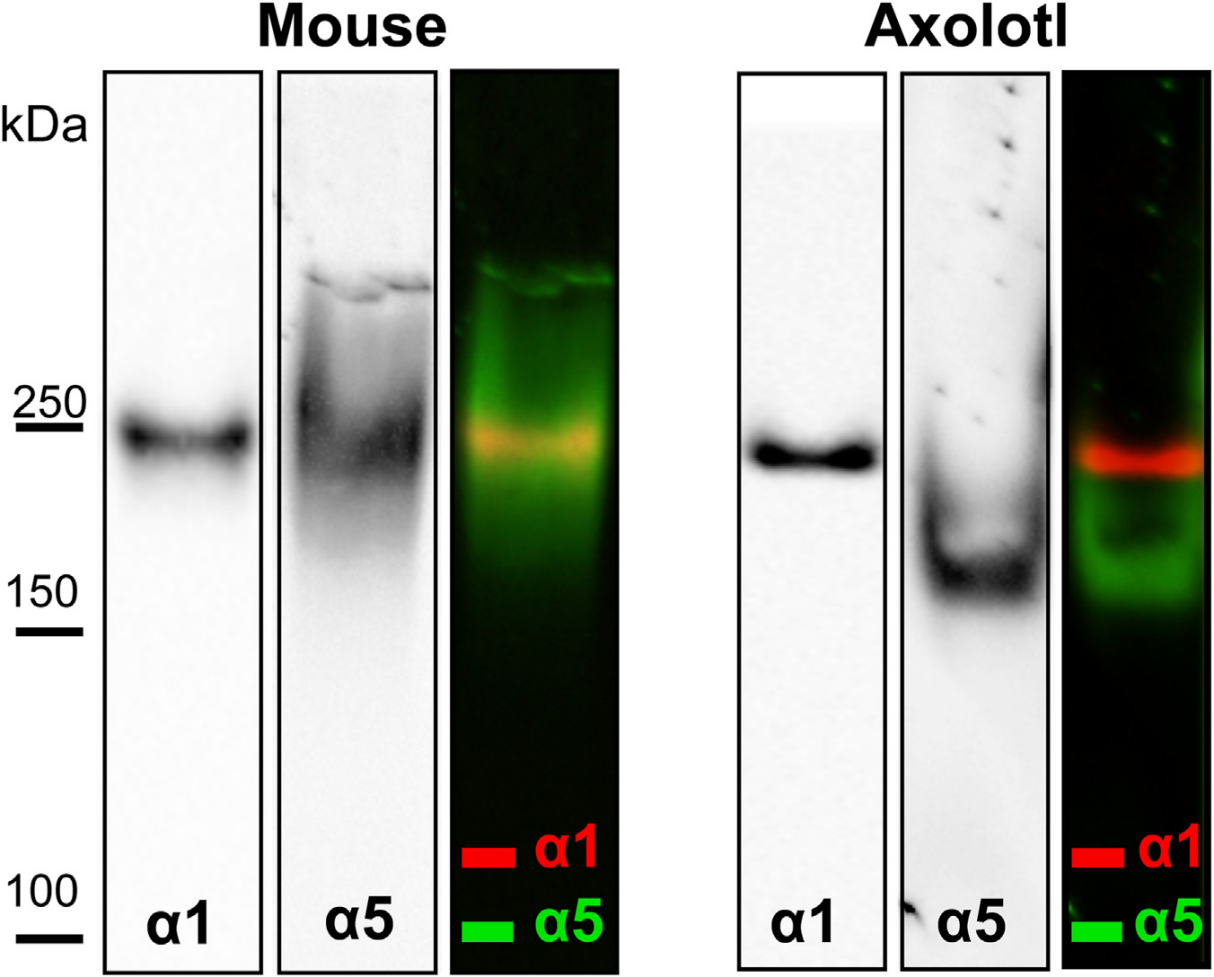
**Col-IV α1 and α5 chains in amphibian kidneys comprise different hexamers.** Two consecutive western blots with α1- and α5-specific antibodies of hexamers prepared from mouse (*left*) and axolotl (*right*) kidneys separated by native PAGE. Third panel for each animal represents overlaid and artificially colored images. The α1 and α5 containing hexamers from axolotl kidneys run as two distinct bands while some overlap, likely from α112-α556 hexamer, is observed in the mouse kidney.

To explore the scaffold composition of chains in the axolotl kidney, we characterized the NC1 hexamers using native PAGE analysis as described for shark kidney *vide supra*. Hexamers were separated on the native page followed by two consecutive western blots with antibodies specific for α1 and α5 chains of collagen IV. Consecutive western blots were performed on the same membrane with a stripping step in between. We then compared the positions of the hexamer bands positive for α1 and α5 chains by overlaying the western blots. Figure 8 represents western blots for each chain and overlaid, artificially colored images. In axolotl, the α1 and α5 containing NC1 hexamers run as two distinctly separate bands. This separation is distinct from that of the mouse kidney. Our results show that α5 and α1 chains exist in different hexamers in axolotl kidneys reflecting a distinction in scaffold compositions between amphibians and mammals. Mice and humans have two α5 containing hexamers (α345-α345 and α112-α556) and two α1 containing hexamers (α112-α112 and α112-α556) in kidneys (Fig. 1) (42, 56, 57, 58, 59). Our results demonstrate the absence of the α112-α556 hexamer in amphibians. Thus, amphibian GBM, devoid of α3 and α4 chains, can potentially have scaffolds composed of previously described combinations, such as α112-α112 and α556-α556. However, the existence of alternative scaffolds such as α555-α555 and α111-α111 cannot be excluded.

### The chain composition of Col-IV scaffolds enabled the morphology of the glomerular basement membrane

**Figure 9:**
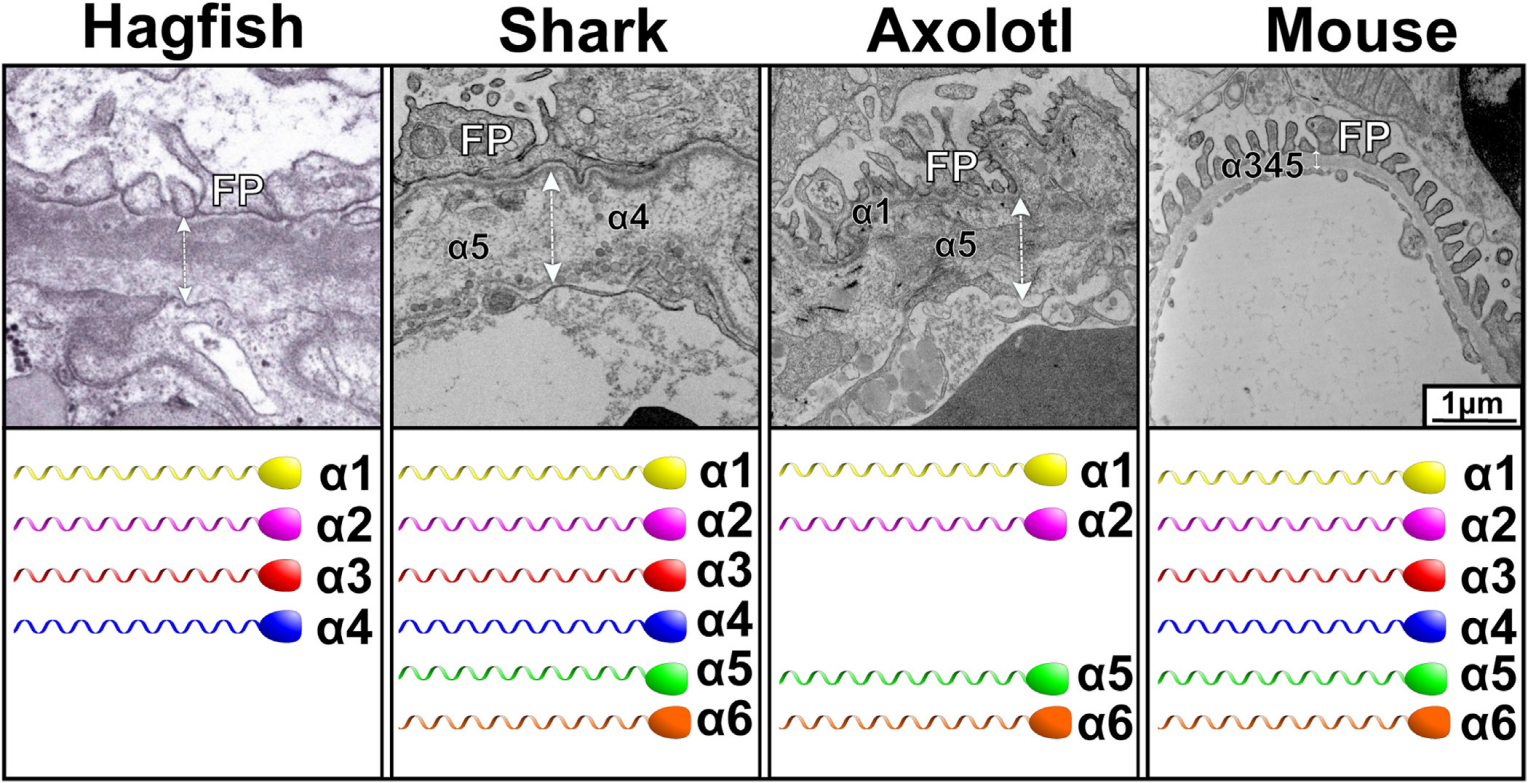
**Comparative morphology of glomerular filtration barrier with Col-IV chains in hagfish, shark, axolotl, and mouse.** Representative TEM images of the glomerular capillary loops from hagfish, shark, axolotl, and mouse are shown, along with the Col IV chains. **FP** designates foot processes of podocytes. *Double sided arrows* indicate GBM (extracellular matrix spanning the space between podocytes and endothelial cells). GBM is variable in thickness, measuring several micrometers in hagfish, shark, and axolotl compared to about 200 nm thick GBM in the mouse. Hagfish displays two-layered organization: equally wide electron dense and electron lucent portions. In shark and axolotl, thin electron dense layer is underlying the podocytes. The rest of GBM is composed of wide electron lucent layer of loose, nonuniform matrix with multiple inclusions of cellular and fibrillar fragments. In the shark thin dense layer is also observed above endothelial cells. In contrast, mouse GBM has smooth and uniform appearance. GBM, glomerular basement membrane.

We explored the ultrastructural morphology of glomerular basement membrane in vertebrate evolution. Figure 9 shows comparative TEM images of the glomerular capillary loops in hagfish, shark, axolotl, and mouse along with the Col-IV chains in each animal. In hagfish, shark, and axolotl, the spacing between the podocytes and endothelial cells of the capillary loop appears strikingly different from that of mouse. Specifically, the GBMs in all early evolutionary species are variable in thickness, ranging from one to several micrometers, and composed of several layers of varying electron density and structural organization. (Fig. 9). Hagfish displays a two-layered organization of the filtration matrix with a more electron dense portion adjacent to podocytes and a more electron translucent subendothelial portion of approximately equal thickness. In shark and axolotl, subpodocyte layer is relatively electron dense and thin compared to the loose, sponge-like, porous subendothelial matrix. In the shark, there is also a thin, electron dense layer adjacent to the endothelial cells. Notably, inclusions of fibrillar fragments are often seen in the electron translucent portion of the GBM in amphibian and shark. In contrast, mammalian GBM is uniform and compact. Thus, there is an evolutionary transition of GBM morphology from hagfish to mammals.

As we demonstrated, the chain composition of kidney Col-IV scaffolds differs among hagfish, shark, amphibians, and mammals (Figure 2, Figure 3, Figure 4, Figure 5, Figure 6, Figure 7, Figure 8). Thus, we explored whether chain composition correlates with the ultrastructural morphology of GBM. As we described *vide supra*, the thin subpodocyte layer in the axolotl is positive for the α1 collagen IV chain, while the loosely structured subendothelial space is predominantly composed of the α5 collagen IV chain. Despite the expression of all six Col-IV chains in shark kidneys (as we demonstrated in the companion paper by *McCaw et al.* (50)), here we showed that major components of GBM, α4 and α5 chains, belong to two different hexamers excluding the existence of Col-IV^α345^ scaffold. Indeed, the ultrastructure of shark’s GBM is more similar to hagfish and axolotl than to mammals. These findings suggest that the emergence of the Col-IV^α345^ scaffold correlates with a morphological transition of the GBM into a compact structure in mammals.

## Discussion

**Figure 10:**
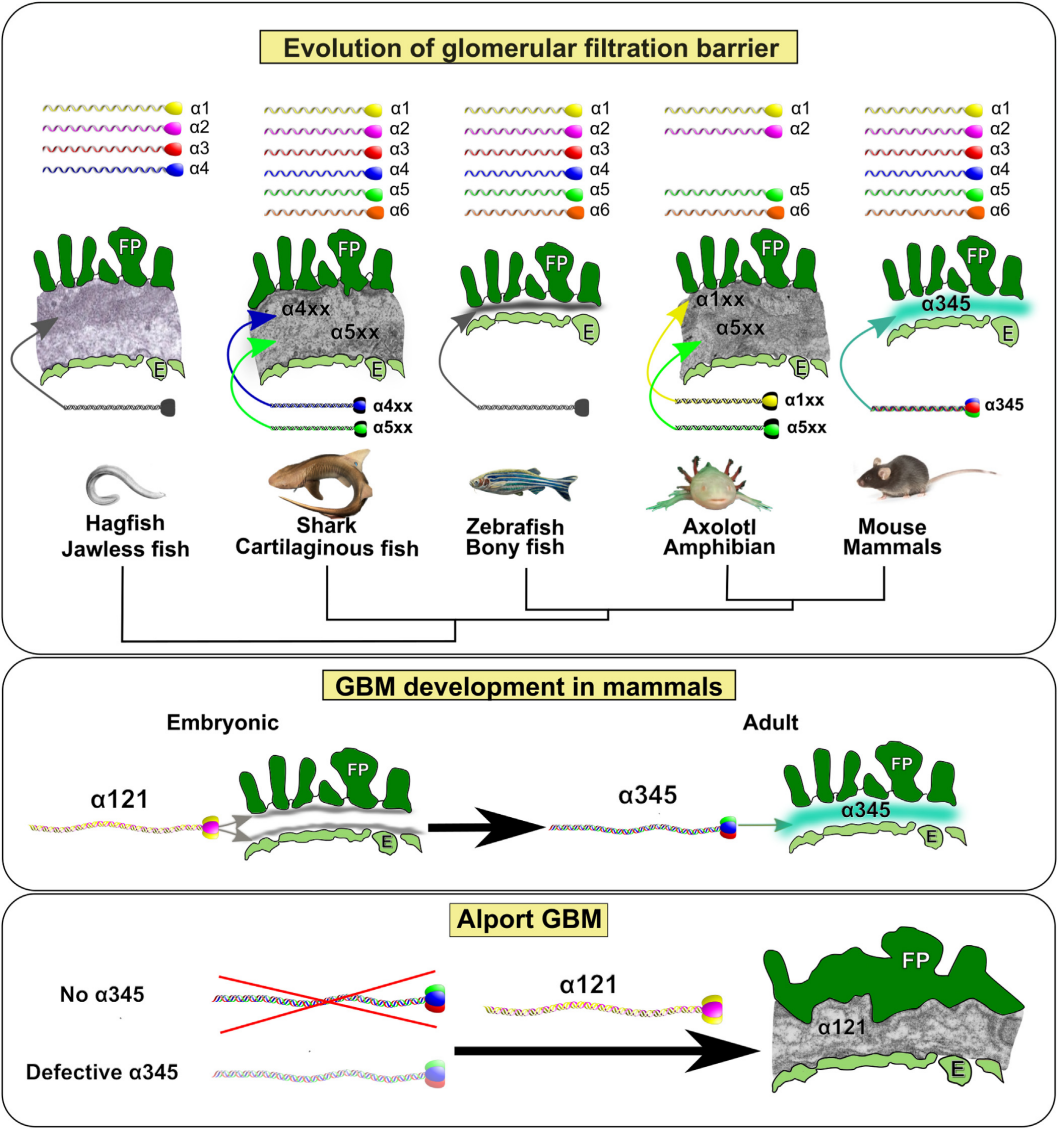
**Col-IV chain composition and structure of the GBM in evolution; development; and disease.***Top panel*. Phylogenetic tree represents animals at the emergence of kidney to mammals along with the number of collagen IV chains and schematic representation of GBM morphology and chain composition. *Middle panel*. The development of mammalian GBM with the switch between α121 and predominant α345 scaffolds is shown schematically. *Bottom panel*. Schematic representation of characteristic GBM abnormalities in Alport Syndrome in the absence of or with defective α345 protomer. GBM, glomerular basement membrane.

Here, we characterized GBM evolution regarding its ultrastructural morphology and collagen IV chain composition. Hagfish, shark, and amphibians have GBM morphologies and Col-IV chain compositions that are distinct from one another and mammals (Fig. 10). GBM morphology evolved from a thick and loosely arranged extracellular matrix in hagfish and shark to a uniform and compact matrix in bony fish (60) and mammals. The α3 and α4 chains emerged first in the hagfish kidney. Subsequently, the α5 chain emerged in the shark kidney along with the α3 and α4 chains, but did not assemble into a Col-IV^α345^ scaffold (Figs. 2 and 4). In the amphibian kidney, the α3 and α4 chains were lost, revealing that the GBM is devoid of the Col-IV^α345^ scaffold but has an α5-containing scaffold. In contrast, the Col-IV^α345^ scaffold is the principal component of mammalian GBM. Collectively, these findings indicate that the assembly of a uniform and compact GBM in mammals was enabled by the evolutionary emergence of the Col-IV^α345^ scaffold.

This enabling role of a scaffold in GBM morphology is supported by a morphologic change in GBM during glomerular development in rodents. Neonates show a dual basement membrane between endothelial cells and podocytes in an early stage of the glomerular capillary loop. As the glomeruli mature, this dual basement membrane fuses and becomes a single, condensed GBM shared between differentiated endothelial cells and podocytes (41, 43, 44, 61). During this maturation process and GBMs fusion, the early Col IV^α121^ scaffold is replaced with Col-IV^α345^ (41, 44) which is derived solely from podocytes (Fig. 10) (62). Thus, the Col-IV^α345^ scaffold coalesces the morphology of developing GBM into a uniform and compact structure.

**Figure 11:**
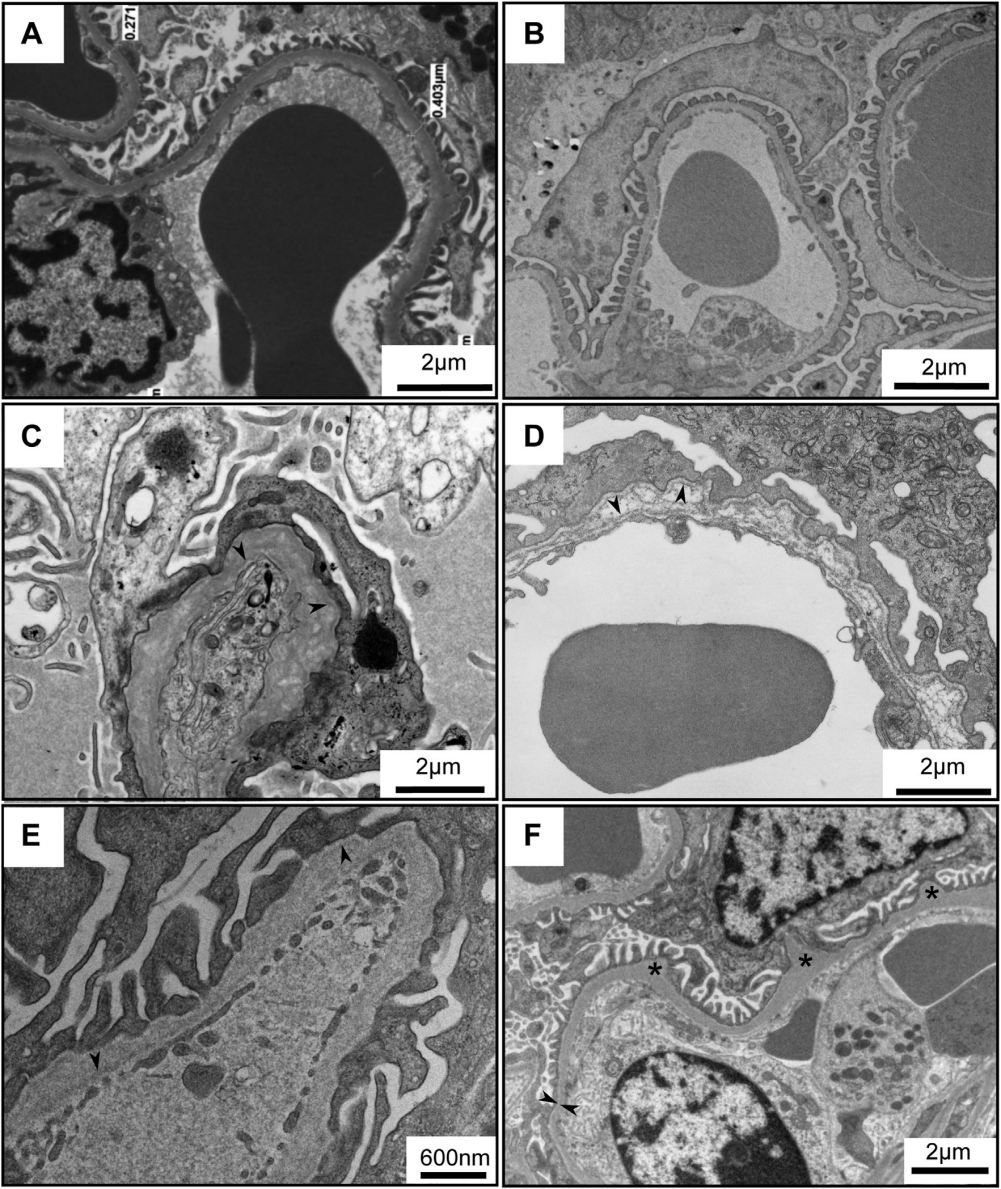
**TEM images of pathological changes in GBM in diseases.***A* and *B*, normal GBM in human (*A*) and in mice (*B*) are compact and uniform. *C* and *D*, a classical lamellation and basketweaving of GBM in a human Alport case (*C*) and *col4a3* KO mouse model (*D*) is presented. *E*, shows abnormal GBM of homozygous Zurich mouse bearing eight amino acid appendage at the C terminus of the α3 chain of Col-IV. This variant also results in a nonuniform, split, and wide GBM. *F*, demonstrates alterations in the GBM of P3H2/GPVI double knockout mouse where a few 3-hydroxylations of prolines are missing. *Arrowheads* in *C*, *D*, and *E* point to thin subpodocyte and subendothelial layers that are spaced by an electron translucent layer; in *F arrowheads* show thinning of GBM. “**∗**” in *F* indicates bulging of GBM. GBM, glomerular basement membrane.

Furthermore, abnormalities in GBM morphology, caused by genetic variants in the Col-IV^α345^ scaffold, provide additional evidence for the role of the scaffold in GBM assembly. The abnormalities were reported previously by numerous investigators. In human Alport syndrome, the GBM is split and laminated compared to normal GBM (Fig. 11, *A* and *C*). This phenotype is copied with knockout *col4a3* mice that are devoid of the Col-IV^α345^ scaffold (Fig. 11*D* compared to control in Fig. 11*B*) (63). Morphology is also affected by the incorporation of the defective scaffold, as in the example of the Z-mouse, wherein an eight amino acid appendage is attached at the C terminus of collagen IV α3 chain (Fig. 11*E*) (45). Therefore, in patients and the mouse models, the absence or defective Col-IV^α345^ scaffold results in irregular thickening, splitting, and prominent visible “layers” in the GBM (arrowheads in Fig. 11, *C*–*E*). Furthermore, even slight changes in posttranslational modifications of the Col-IV^α345^ scaffold, such as the absence of a few 3-hydroxylations of prolines in the collagen IV of P3H2 knockout mouse, causes irregular thinning and bulging of the GBM (Fig. 11*F*) (64). Collectively, these findings pinpoint a critical role of the Col-IV^α345^ scaffold enabling a uniform and compact GBM.

In mammals, the compact GBM functions as a permselective filter that restricts the passage of protein from circulating blood into the glomerular filtrate (12). The evidence is based on several lines of research from multiple labs over 6 decades, beginning in the 1960s. Tracers injected intravenously into rats showed the normal but not nephrotic GBM to be impermeable to ferritin, a large electron-dense particle (51, 65). Likewise, mouse GBM is impermeable to ferritin, whereas the GBM of an Alport mouse, devoid of the Col-IV^α345^ scaffold, is freely permeable (66). Genetic variants (Fig. 11) of the Col-IV^α345^ scaffold in humans and in mouse models cause a broad spectrum of phenotypes, ranging from microscopic hematuria to progressive proteinuria and end-stage kidney failure (5, 15, 67). Moreover, the GBM of laminin-521 knockout mice is freely permeable to ferritin in contrast to control littermates (35, 68). Collectively, the findings reveal that GBM functions as an ultrafilter of proteins and pinpoint a critical role of the Col-IV^α345^ scaffold and laminin-521 in the filtration mechanism.

Yet, the fundamental question of how Col-IV^α345^ and laminin-521 confer a filtration function to the GBM remains an enigma. Variants in Col-IV^α345^ lead to progressive proteinuria, whereas laminin-521 variants cause early proteinuria. Tracer studies in laminin-521 knockout mice show ferritin permeability across the width of the GBM (35), indicating that the laminin network is closely associated with the Col-IV^α345^ scaffold. The Col-IV^α345^ scaffold is presumed to tether the laminin-521 network, along with other macromolecules constituents, forming a supramolecular complex that functions as an ultrafilter of proteins (Fig. 1). Evidence is emerging that this permselective function of GBM is linked to the compression of this supramolecular complex by physical forces of hydrostatic pressure in the glomerular capillaries. In disease, reduced compression of the GBM complex allows the passage of albumin (6, 69, 70). Solving the ultrafiltration mystery hinges on gaining new knowledge of the supramolecular structure and assembly and how each GBM component functions at molecular and atomic levels. Future pursuits of primary defects that cause proteinuria in other glomerular diseases, as in diabetic nephropathy and focal segmental glomerulosclerosis, may lead to discoveries of new glomerular proteins (71), as well as elucidation of the molecular functionality of Col-IV^α345^, laminin-521 and other currently known GBM components.

In conclusion, our findings prompted us to revisit the conundrum, defined 4 decades ago, as to the nature of the primary protein filter. Farquhar *et al.* concluded from ferritin tracer studies in rats that the GBM is the primary filter (51). In opposition, Rodewald *et al.* concluded from ferritin tracer studies in frog (52), and later by Tanner *et al.* on salamander (53), that the slit diaphragm between the podocytes is the main filtration barrier. Numerous subsequent studies in mice provided evidence in support of both GBM (35, 51, 66, 72, 73) and slit diaphragm as filters (37, 72, 74). Herein, we found that amphibian GBM is devoid of Col-IV^α345^, thus explaining the early conclusion that the slit diaphragm and not the GBM is the ultrafilter. In contrast, GBM of rat and mouse is composed of the Col-IV^α345^ scaffold, explaining the GBM impermeability to ferritin.

Thus, both groups were correct. Moreover, the concluding statement by Schaffner and Rodewald in the study of bullfrog, was insightful: “It is possible that a shift in barrier function from the slit diaphragm in bullfrog to an increasingly less permeable basement membrane in rats may have occurred during evolution of a more efficient glomerular filter” (52). Our findings, using an evolutionary approach, revealed that it was the evolutionary emergence of the Col-IV^α345^ scaffold that enabled the genesis of a compact GBM in mammals that functions as an ultrafilter of proteins. Perhaps, the heavily crosslinked Col-IV^α345^ scaffold (Fig. 1) conferred tensile strength to resist the high glomerular capillary pressure in mammals, whereas the pressure in lower vertebrates is low (75, 76), requiring only the slit diaphragm as a filter. Collectively, the present evidence favors the GBM as an initial ultrafilter of proteins in mammals, and the slit diaphragm as a sequential filter in the glomerular filtration barrier of mammals.

## Experimental procedures

### Animals husbandry

#### Axolotls (A. mexicanum)

Used in experiments were either purchased from Ambystoma genomic stock center or bred in MDIBL facilities. Animals were housed in aquatic tank with a room temperature of 20 °C and 12-h day/light cycle. For the detailed husbandry conditions, please refer to the previous publication (77). Animal experiments were performed as approved by the IACUC committee of MDIBL, and all animal handling and surgical procedures were carried out in accordance with local ethics committee guidelines. “White” refers to a nontransgenic d/d strain of axolotl that has white skin due to the absence of melanocytes. Animals were anesthetized in 0.03% benzocaine (Sigma) before amputation and surgery.

#### Nurse shark, *Ginglymostoma cirratum*

Elasmobranch, cartilaginous fish (Chondrichthyes) named “Tim” used in this study was an adult healthy male of approximately 12 years old, originally caught wild near the Florida Keys and maintained in captivity in the aquarium in Baltimore, MD, for multiple years. All procedures were performed as approved by the University of Maryland Baltimore IACUC committee.

#### Frogs, *X. laevis*

Used in the study were housed at Vanderbilt in an AAALAC-approved amphibian satellite facility.

#### Mice

Used in the study were housed at Vanderbilt. The C57/B6 mouse strain was used as wildtype. Mouse and frog experiments were performed as approved by the Vanderbilt’s IACUC committee, and all animal handling and procedures were carried out in accordance with local ethics committee guidelines.

### Chain-specific antibodies

The following chain-specific primary antibodies were used: H11 (α1); H31 (α3); H43 (α4); and Mab5 (α5). Rat anti-human monoclonal antibodies H11, H31, and H43 were from Chondrex (55). Mouse anti-bovine monoclonal antibody Mab5 was from Wieslab.

### Immunofluorescent staining of frozen kidney sections

Kidneys from mouse, shark, axolotl, and frog were snap frozen in OCT immediately after isolation. Cryostat (Leica CM 1950) was used to cut 7 μm sections. Sections were mounted on glass slides, air dried for 15 to 30 min at room temperature, and fixed in acetone at −20 ^°^C for 10 min. After three washes with 50 mM Tris, 150 mM NaCl, 0.1% Tween-20 buffer (TBS-t) denaturation step was performed. Namely, tissues sections were incubated in acid urea for 20 min at room temperature (25 ^°^C) (6M urea in 0.1 M glycine, pH 3.0). Sections were washed 3 × 5 min with TBS-t. Blocking was performed with 10% goat serum (Invitrogen, 50062Z) for 1 h. Sections were incubated with primary antibodies (generally 1:1000 dilution in 1% goat serum TBS-t) overnight in the cold room. Following 3 × 15 min washes with TBS-t, secondary goat anti-rat or anti-mouse antibodies conjugated with fluorescent tag were incubated on sections for 1.5 h at room temperature. Sections were then washed and mounted with antifade mounting solution with DAPI. Images were taken with Nikon Eclipse *Ti* microscope and analyzed with GIMP.

### Double immunofluorescent staining

Double immunofluorescent staining was performed using the following pairs of antibodies: H11 (α1) and Mab5 (α5) for amphibians and H43 and Mab5 for shark. Secondary antibodies were goat anti-rat-Alexa568 (red) and goat anti-mouse-Alexa488 (green) (Thermo Fisher Scientific).

### Western blots and collagen IV chain-specific antibodies

Western blotting was done following standard technique described elsewhere (78) using HRP-conjugated secondary antibodies (Thermo Fisher Scientific). All Western blotting of native and SDS-dissociated NC1 hexamers was done with ThermoScientific SuperSignal West Femto chemiluminescent substrate and digitally imaged with a Bio-Rad GelDoc. For all western blots including native gels, the same set of antibodies as for immunofluorescence was used. Stripping step was done with Restore stripping buffer (Thermo Fisher Scientific). Native PAGE electrophoresis was performed as previously described with gels made in house (79).

### Double ISEM

Frozen kidney sections were placed on 22 mm round plastic tissue culture cover slips (Sarstedt, Inc). Double immunolabeling was performed with H11 (α1) and Mab5 (α5) antibodies following the same protocol as described for immunofluorescence but with no initial fixation step. Negative controls with no primary antibodies for secondary immunogold-conjugated antibodies were performed. Secondary anti-rat and anti-mouse antibodies conjugated with 6 nm and 15 nm gold particles, respectively, were used (EMS). Immunolabeled samples went through standard protocol for SEM preparation, including postfixation in 1% osmium tetroxide (reduced to 20 min), serial dehydration, and critical point drying (EMS 850). Finally, samples were carbon coated for 1 s on Electron Microscopy Sciences carbon coater (EMS 950x). Images were taken with Quanta 250 Environmental Scanning Electron Microscope in secondary and backscattering modes. For immunogold-labeled samples, the images produced by secondary and backscattered electrons were overlaid, and gold particles were artificially colored in GIMP (The GIMP Development Team, 2023. *GIMP*, Available at: https://www.gimp.org).

### Transmission electron microscopy

Fresh tissues were fixed in 2.5% glutaraldehyde buffered in 0.1 M sodium cacodylate buffer, pH ∼7.5, postfixed in 1% osmium tetroxide, followed by dehydration through a grade series of ethanol to 100%. Samples were further dehydrated in propylene oxide and infiltrated and embedded in Spurr’s epoxy. 70-nm ultrathin sections were collected on 300 mesh copper grids and stained in 2% uranyl acetate followed by Reynold’s lead citrate. Stained sections were examined using a T-12 electron microscope (Philips/FEI) operated at 100 kV and photographed using a 2K camera (AMT). Samples embedding and sectioning was done by Cell Imaging Vanderbilt University Core.

## Data availability

All data are contained within the manuscript. Raw SEM images from this study are available by emailing the corresponding author.

## Conflict of interest

The authors declare no conflict of interest with the contents of this article.

## Footnotes

† GBM is composed of 86% protein, 9% carbohydrate and <1% lipid of dry weight, based on chemical analyses. The high glycine and hydroxylysine contents revealed that GBM is comprised of an unknown type of collagen; at the time, only collagen types I and II were known. Subsequently, the GBM collagen was identified as collagen IV and shown to exists in the glomerulus in two forms: Col-IV ^α121^ and Col-IV ^α345^ scaffolds (Fig. 1). These structures tether proteins including laminin, nidogen and proteoglycans and other components, forming a supramolecular complex (Fig. 1). In glomerular development, fetal Col-IV ^α121^ in GBM is replaced by Col-IV ^α345^ in adult, based on studies of mouse, human, and dogs. The relative amount of Col-IV ^α345^ in adult GBM is 74%, a value based on the glycine content of whole bovine GBM and that of the mouse α3, α4, and α5 chains of the Col-IV ^α345^ scaffold (207 residues/1000 residues *versus* 278, respectively).

## References

[1] Boudko S.P., Pedchenko V.K., Pokidysheva E.N., Budko A.M., Baugh R., Coates P.T. (2023). Collagen IV of basement membranes: III. Chloride pressure is a primordial innovation that drives and maintains the assembly of scaffolds. J. Biol. Chem..

[2] Kovesdy C.P. (2022). Epidemiology of chronic kidney disease: an update 2022. Kidney Int. Suppl. (2011).

[3] Levin A., Tonelli M., Bonventre J., Coresh J., Donner J.A., Fogo A.B. (2017). Global kidney health 2017 and beyond: a roadmap for closing gaps in care, research, and policy. Lancet.

[4] Groopman E.E., Marasa M., Cameron-Christie S., Petrovski S., Aggarwal V.S., Milo-Rasouly H. (2019). Diagnostic utility of exome sequencing for kidney disease. N. Engl. J. Med..

[5] Kashtan C.E., Ding J., Garosi G., Heidet L., Massella L., Nakanishi K. (2018). Alport syndrome: a unified classification of genetic disorders of collagen IV alpha345: a position paper of the alport syndrome classification working group. Kidney Int..

[6] Benzing T., Salant D. (2021). Insights into glomerular filtration and albuminuria. N. Engl. J. Med..

[7] Chen T.K., Knicely D.H., Grams M.E. (2019). Chronic kidney disease diagnosis and management: a review. JAMA.

[8] Savige J. (2014). Alport syndrome: its effects on the glomerular filtration barrier and implications for future treatment. J. Physiol..

[9] Zandi-Nejad K., Eddy A.A., Glassock R.J., Brenner B.M. (2004). Why is proteinuria an ominous biomarker of progressive kidney disease?. Kidney Int. Suppl..

[10] Palmer B.F. (2007). Proteinuria as a therapeutic target in patients with chronic kidney disease. Am. J. Nephrol..

[11] Iseki K., Ikemiya Y., Iseki C., Takishita S. (2003). Proteinuria and the risk of developing end-stage renal disease. Kidney Int..

[12] Scott R.P., Quaggin S.E. (2015). Review series: the cell biology of renal filtration. J. Cell Biol..

[13] Gheith O., Farouk N., Nampoory N., Halim M.A., Al-Otaibi T. (2016). Diabetic kidney disease: world wide difference of prevalence and risk factors. J. Nephropharmacol..

[14] Anders H.J., Huber T.B., Isermann B., Schiffer M. (2018). CKD in diabetes: diabetic kidney disease versus nondiabetic kidney disease. Nat. Rev. Nephrol..

[15] Gibson J., Fieldhouse R., Chan M.M.Y., Sadeghi-Alavijeh O., Burnett L., Izzi V. (2021). Prevalence estimates of predicted pathogenic col4a3-COL4A5 variants in a population sequencing database and their implications for Alport syndrome. J. Am. Soc. Nephrol..

[16] Matejas V., Al-Gazali L., Amirlak I., Zenker M. (2006). A syndrome comprising childhood-onset glomerular kidney disease and ocular abnormalities with progressive loss of vision is caused by mutated LAMB2. Nephrol. Dial. Transpl..

[17] Zenker M., Aigner T., Wendler O., Tralau T., Muntefering H., Fenski R. (2004). Human laminin beta2 deficiency causes congenital nephrosis with mesangial sclerosis and distinct eye abnormalities. Hum. Mol. Genet..

[18] Matejas V., Hinkes B., Alkandari F., Al-Gazali L., Annexstad E., Aytac M.B. (2010). Mutations in the human laminin beta2 (LAMB2) gene and the associated phenotypic spectrum. Hum. Mutat..

[19] Keskar V., Jamale T.E., Kulkarni M.J., Kiggal Jagadish P., Fernandes G., Hase N. (2013). Minimal-change disease in adolescents and adults: epidemiology and therapeutic response. Clin. Kidney J..

[20] Keri K.C., Blumenthal S., Kulkarni V., Beck L., Chongkrairatanakul T. (2019). Primary membranous nephropathy: comprehensive review and historical perspective. Postgrad. Med. J..

[21] Bajema I.M., Balow J.E., Haas M., Jayne D., Lightstone L., Rovin B.H. (2023). Update on scoring and providing evidence basis for assessing pathology in lupus nephritis. Kidney Int..

[22] Almaani S., Meara A., Rovin B.H. (2017). Update on lupus nephritis. Clin. J. Am. Soc. Nephrol..

[23] Sambharia M., Rastogi P., Thomas C.P. (2022). Monogenic focal segmental glomerulosclerosis: a conceptual framework for identification and management of a heterogeneous disease. Am. J. Med. Genet. C Semin. Med. Genet..

[24] Kestila M., Lenkkeri U., Mannikko M., Lamerdin J., McCready P., Putaala H. (1998). Positionally cloned gene for a novel glomerular protein--nephrin--is mutated in congenital nephrotic syndrome. Mol. Cell.

[25] Khoshnoodi J., Tryggvason K. (2001). Congenital nephrotic syndromes. Curr. Opin. Genet. Dev..

[26] Farquhar M.G. (2006). The glomerular basement membrane: not gone, just forgotten. J. Clin. Invest..

[27] Wieslander J., Barr J.F., Butkowski R.J., Edwards S.J., Bygren P., Heinegard D. (1984). Goodpasture antigen of the glomerular basement membrane: localization to noncollagenous regions of type IV collagen. Proc. Natl. Acad. Sci. U. S. A..

[28] Derry C.J., Pickering M., Baker C., Pusey C.D. (1994). Identification of the Goodpasture antigen, alpha 3(IV) NC1, and four other NC1 domains of type IV collagen, by amino-terminal sequence analysis of human glomerular basement membrane separated by two-dimensional electrophoresis. Exp. Nephrol..

[29] Saus J., Wieslander J., Langeveld J.P., Quinones S., Hudson B.G. (1988). Identification of the Goodpasture antigen as the alpha 3(IV) chain of collagen IV. J. Biol. Chem..

[30] Butkowski R.J., Langeveld J.P., Wieslander J., Hamilton J., Hudson B.G. (1987). Localization of the goodpasture epitope to a novel chain of basement membrane collagen. J. Biol. Chem..

[31] Hudson B.G. (2004). The molecular basis of goodpasture and alport syndromes: beacons for the discovery of the collagen IV family. J. Am. Soc. Nephrol..

[32] Hudson B.G., Reeders S.T., Tryggvason K. (1993). Type IV collagen: structure, gene organization, and role in human diseases. Molecular basis of goodpasture and alport syndromes and diffuse leiomyomatosis. J. Biol. Chem..

[33] Barker D.F., Hostikka S.L., Zhou J., Chow L.T., Oliphant A.R., Gerken S.C. (1990). Identification of mutations in the COL4A5 collagen gene in Alport syndrome. Science.

[34] Hostikka S.L., Eddy R.L., Byers M.G., Hoyhtya M., Shows T.B., Tryggvason K. (1990). Identification of a distinct type IV collagen alpha chain with restricted kidney distribution and assignment of its gene to the locus of X chromosome-linked Alport syndrome. Proc. Natl. Acad. Sci. U. S. A..

[35] Jarad G., Cunningham J., Shaw A.S., Miner J.H. (2006). Proteinuria precedes podocyte abnormalities inLamb2-/- mice, implicating the glomerular basement membrane as an albumin barrier. J. Clin. Invest..

[36] Hinkes B.G., Mucha B., Vlangos C.N., Gbadegesin R., Liu J., Hasselbacher K. (2007). Nephrotic syndrome in the first year of life: two thirds of cases are caused by mutations in 4 genes (NPHS1, NPHS2, WT1, and LAMB2). Pediatrics.

[37] Tryggvason K., Patrakka J., Wartiovaara J. (2006). Hereditary proteinuria syndromes and mechanisms of proteinuria. N. Engl. J. Med..

[38] Beck L.H., Bonegio R.G., Lambeau G., Beck D.M., Powell D.W., Cummins T.D. (2009). M-type phospholipase A2 receptor as target antigen in idiopathic membranous nephropathy. N. Engl. J. Med..

[39] Hudson B.G., Spiro R.G. (1972). Studies on the native and reduced alkylated renal glomerular basement membrane. Solubility, subunit size, and reaction with cyanogen bromide. J. Biol. Chem..

[40] Spiro R.G. (1967). Studies on the renal glomerular basement membrane. Preparation and chemical composition. J. Biol. Chem..

[41] Miner J.H., Sanes J.R. (1994). Collagen IV alpha 3, alpha 4, and alpha 5 chains in rodent basal laminae: sequence, distribution, association with laminins, and developmental switches. J. Cell Biol..

[42] Gunwar S., Ballester F., Noelken M.E., Sado Y., Ninomiya Y., Hudson B.G. (1998). Glomerular basement membrane. Identification of a novel disulfide-cross-linked network of alpha3, alpha4, and alpha5 chains of type IV collagen and its implications for the pathogenesis of Alport syndrome. J. Biol. Chem..

[43] Kalluri R., Shield C.F., Todd P., Hudson B.G., Neilson E.G. (1997). Isoform switching of type IV collagen is developmentally arrested in X-linked Alport syndrome leading to increased susceptibility of renal basement membranes to endoproteolysis. J. Clin. Invest..

[44] Harvey S.J., Zheng K., Sado Y., Naito I., Ninomiya Y., Jacobs R.M. (1998). Role of distinct type IV collagen networks in glomerular development and function. Kidney Int..

[45] Pokidysheva E.N., Seeger H., Pedchenko V., Chetyrkin S., Bergmann C., Abrahamson D. (2021). Collagen IV(alpha345) dysfunction in glomerular basement membrane diseases. I. Discovery of a COL4A3 variant in familial Goodpasture's and Alport diseases. J. Biol. Chem..

[46] Heidet L., Arrondel C., Forestier L., Cohen-Solal L., Mollet G., Gutierrez B. (2001). Structure of the human type IV collagen gene COL4A3 and mutations in autosomal Alport syndrome. J. Am. Soc. Nephrol..

[47] Mochizuki T., Lemmink H.H., Mariyama M., Antignac C., Gubler M.C., Pirson Y. (1994). Identification of mutations in the alpha 3(IV) and alpha 4(IV) collagen genes in autosomal recessive Alport syndrome. Nat. Genet..

[48] Savige J., Huang M., Croos Dabrera M.S., Shukla K., Gibson J. (2022). Genotype-phenotype correlations for pathogenic col4a3-COL4A5 variants in X-linked, autosomal recessive, and autosomal dominant Alport syndrome. Front. Med. (Lausanne).

[49] Suzuki R., Sakakibara N., Ichikawa Y., Kitakado H., Ueda C., Tanaka Y. (2023). Systematic review of clinical characteristics and Genotype-phenotype correlation in LAMB2-associated disease. Kidney Int. Rep..

[50] Page-McCaw P.S., Pokidysheva E.N., Darris C.E., Chetyrkin S., Murawala P., Gallup J. (2023). Collagen IV of basement membranes: I. Origin and diversification of COL4 genes enabling animal evolution. bioRxiv.

[51] Farquhar M.G., Wissig S.L., Palade G.E. (1961). Glomerular permeability. I. Ferritin transfer across the normal glomerular capillary wall. J. Exp. Med..

[52] Schaffner A., Rodewald R. (1978). Glomerular permeability in the bullfrog Rana catesbeiana. J. Cell Biol..

[53] Tanner G.A., Rippe C., Shao Y., Evan A.P., Williams J.C. (2009). Glomerular permeability to macromolecules in the Necturus kidney. Am. J. Physiol. Renal Physiol..

[54] Boutaud A., Borza D.B., Bondar O., Gunwar S., Netzer K.O., Singh N. (2000). Type IV collagen of the glomerular basement membrane. Evidence that the chain specificity of network assembly is encoded by the noncollagenous NC1 domains. J. Biol. Chem..

[55] Ninomiya Y., Kagawa M., Iyama K., Naito I., Kishiro Y., Seyer J.M. (1995). Differential expression of two basement membrane collagen genes, COL4A6 and COL4A5, demonstrated by immunofluorescence staining using peptide-specific monoclonal antibodies. J. Cell Biol..

[56] Robertson W.E., Rose K.L., Hudson B.G., Vanacore R.M. (2014). Supramolecular organization of the alpha121-alpha565 collagen IV network. J. Biol. Chem..

[57] Vanacore R.M., Ham A.J., Cartailler J.P., Sundaramoorthy M., Todd P., Pedchenko V. (2008). A role for collagen IV cross-links in conferring immune privilege to the goodpasture autoantigen: structural basis for the crypticity of B cell epitopes. J. Biol. Chem..

[58] Boudko S.P., Bauer R., Chetyrkin S.V., Ivanov S., Smith J., Voziyan P.A. (2021). Collagen IV(alpha345) dysfunction in glomerular basement membrane diseases. II. Crystal structure of the alpha345 hexamer. J. Biol. Chem..

[59] Borza D.B., Bondar O., Ninomiya Y., Sado Y., Naito I., Todd P. (2001). The NC1 domain of collagen IV encodes a novel network composed of the alpha 1, alpha 2, alpha 5, and alpha 6 chains in smooth muscle basement membranes. J. Biol. Chem..

[60] Huang J., McKee M., Huang H.D., Xiang A., Davidson A.J., Lu H.A. (2013). A zebrafish model of conditional targeted podocyte ablation and regeneration. Kidney Int..

[61] Abrahamson D.R. (2009). Development of kidney glomerular endothelial cells and their role in basement membrane assembly. Organogenesis.

[62] Abrahamson D.R., Hudson B.G., Stroganova L., Borza D.B., St John P.L. (2009). Cellular origins of type IV collagen networks in developing glomeruli. J. Am. Soc. Nephrol..

[63] Cosgrove D., Meehan D.T., Grunkemeyer J.A., Kornak J.M., Sayers R., Hunter W.J. (1996). Collagen COL4A3 knockout: a mouse model for autosomal Alport syndrome. Genes Dev..

[64] Aypek H., Krisp C., Lu S., Liu S., Kylies D., Kretz O. (2022). Loss of the collagen IV modifier prolyl 3-hydroxylase 2 causes thin basement membrane nephropathy. J. Clin. Invest..

[65] Farquhar M.G., Palade G.E. (1961). Glomerular permeability. II. Ferritin transfer across the glomerular capillary wall in nephrotic rats. J. Exp. Med..

[66] Abrahamson D.R., Isom K., Roach E., Stroganova L., Zelenchuk A., Miner J.H. (2007). Laminin compensation in collagen alpha3(IV) knockout (Alport) glomeruli contributes to permeability defects. J. Am. Soc. Nephrol..

[67] Pirson Y. (1999). Making the diagnosis of Alport's syndrome. Kidney Int..

[68] Suh J.H., Miner J.H. (2013). The glomerular basement membrane as a barrier to albumin. Nat. Rev. Nephrol..

[69] Fissell W.H., Miner J.H. (2018). What is the glomerular ultrafiltration barrier?. J. Am. Soc. Nephrol..

[70] Butt L., Unnersjo-Jess D., Hohne M., Edwards A., Binz-Lotter J., Reilly D. (2020). A molecular mechanism explaining albuminuria in kidney disease. Nat. Metab..

[71] Lennon R., Byron A., Humphries J.D., Randles M.J., Carisey A., Murphy S. (2014). Global analysis reveals the complexity of the human glomerular extracellular matrix. J. Am. Soc. Nephrol..

[72] Tryggvason K., Pettersson E. (2003). Causes and consequences of proteinuria: the kidney filtration barrier and progressive renal failure. J. Intern. Med..

[73] Abrahamson D.R., St John P.L., Stroganova L., Zelenchuk A., Steenhard B.M. (2013). Laminin and type IV collagen isoform substitutions occur in temporally and spatially distinct patterns in developing kidney glomerular basement membranes. J. Histochem. Cytochem..

[74] Tryggvason K. (1999). Unraveling the mechanisms of glomerular ultrafiltration: nephrin, a key component of the slit diaphragm. J. Am. Soc. Nephrol..

[75] Schulte K., Kunter U., Moeller M.J. (2015). The evolution of blood pressure and the rise of mankind. Nephrol. Dial. Transpl..

[76] Ichimura K., Sakai T. (2017). Evolutionary morphology of podocytes and primary urine-producing apparatus. Anat. Sci. Int..

[77] Khattak S., Murawala P., Andreas H., Kappert V., Schuez M., Sandoval-Guzman T. (2014). Optimized axolotl (Ambystoma mexicanum) husbandry, breeding, metamorphosis, transgenesis and tamoxifen-mediated recombination. Nat. Protoc..

[78] Mahmood T., Yang P.C. (2012). Western blot: technique, theory, and trouble shooting. N. Am. J. Med. Sci..

[79] Arndt C., Koristka S., Feldmann A., Bachmann M. (2019). Native Polyacrylamide gels. Methods Mol. Biol..

[80] Yurchenco P.D., Ruben G.C. (1987). Basement membrane structure *in situ*: evidence for lateral associations in the type IV collagen network. J. Cell Biol..

